# Low Klotho/Fibroblast Growth Factor 23 Ratio Is an Independent Risk Factor for Renal Progression in Chronic Kidney Disease: Finding From KNOW-CKD

**DOI:** 10.3389/fmed.2022.904963

**Published:** 2022-07-08

**Authors:** Hyo Jin Kim, Yunmi Kim, Minjung Kang, Seonmi Kim, Sue Kyung Park, Suah Sung, Young Youl Hyun, Ji Yong Jung, Curie Ahn, Kook-Hwan Oh

**Affiliations:** ^1^Department of Internal Medicine, Pusan National University School of Medicine, Busan, South Korea; ^2^Biomedical Research Institute, Pusan National University Hospital, Busan, South Korea; ^3^Department of Internal Medicine, Inje University Busan Paik Hospital, Busan, South Korea; ^4^Department of Internal Medicine, Seoul National University Hospital, Seoul, South Korea; ^5^Department of Preventive Medicine, Seoul National University College of Medicine, Seoul, South Korea; ^6^Department of Internal Medicine, Eulji Medical Center, Eulji University, Seoul, South Korea; ^7^Department of Internal Medicine, Kangbuk Samsung Hospital, Sungkyunkwan University School of Medicine, Seoul, South Korea; ^8^Department of Internal Medicine, Gachon University Gil Medical Center, Gachon University College of Medicine, Incheon, South Korea; ^9^Department of Internal Medicine, National Medical Center, Seoul, South Korea; ^10^Department of Internal Medicine, Seoul National University College of Medicine, Seoul, South Korea

**Keywords:** Klotho, fibroblast growth factor 23, chronic kidney disease, renal progression, mortality

## Abstract

**Background:**

We aimed to evaluate soluble Klotho and circulating fibroblast growth factor 23 (FGF23) ratio as a risk factor for renal progression, cardiovascular (CV) events, and mortality in chronic kidney disease (CKD).

**Methods:**

We analyzed 2,099 subjects from a CKD cohort whose soluble Klotho and C-terminal FGF23 levels were measured at enrollment. The Klotho to FGF23 ratio was calculated as Klotho values divided by FGF23 values + 1 (hereinafter called the Klotho/FGF23 ratio). Participants were categorized into quartiles according to Klotho/FGF23 ratio. The primary outcome was renal events, defined as the doubling of serum creatinine, 50% reduction of estimated glomerular filtration rate from the baseline values, or development of end-stage kidney disease. The secondary outcomes consisted of CV events and death. Changes in CV parameters at the time of enrollment and during follow-up according to the Klotho/FGF23 ratio were also examined.

**Results:**

During the follow-up period of 64.0 ± 28.2 months, 735 (35.1%) and 273 (13.0%) subjects developed renal events and composite outcomes of CV events and death, respectively. After adjustment, the first (HR: 1.36; 95% CI: 1.08–1.72, *P* = 0.010) and second (HR: 1.45; 95% CI: 1.15–1.83, *P* = 0.002) quartiles with regard to the Klotho/FGF23 ratio showed elevated risk of renal events as compared to the fourth quartile group. There was no significant association between Klotho/FGF23 ratio and the composite outcome of CV events and death. The prevalence of left ventricular hypertrophy and vascular calcification was higher in the low Klotho/FGF23 ratio quartiles at baseline and at the fourth-year follow-up.

**Conclusions:**

Low Klotho/FGF23 ratio was significantly associated with increased renal events in the cohort of Korean predialysis CKD patients.

## Introduction

Klotho and fibroblast growth factor 23 (FGF23) are early laboratory parameters of chronic kidney disease (CKD)-mineral bone disorder (MBD), and the Klotho/FGF23 axis plays an important role in this disorder (1, 2). Klotho, which is an anti-aging protein, is closely associated with CKD, since the kidney is the major organ for the production of Klotho, and CKD is known to be a Klotho-deficient state (3, 4). FGF23 is a phosphorus-regulating protein secreted by bone cells, and serum FGF23 level increases as kidney function declines (5, 6). In most previous studies, low soluble Klotho levels were associated with increased adverse kidney outcomes (7). Among CKD patients, the subjects with lower serum Klotho levels (lower than median: ≤396.3 pg/mL) exhibited poorer outcomes [doubling serum creatinine, end stage kidney disease (ESKD), or death] than those with higher levels (8). A community-based elderly cohort study showed that higher soluble Klotho level was independently associated with a lower risk of decline in kidney function, defined as eGFR decline ≥30% or eGFR decline >3 ml/min per year (9). However, there was also a study in which soluble Klotho was not related to kidney function and did not predict adverse outcomes in CKD patients (10). In addition, previous studies showed that both C-terminal and intact FGF23 independently predicted the progression of CKD after adjustment for multiple factors in patients with non-diabetic CKD (11). Higher FGF23 levels were likely associated with coronary calcification (12, 13) and all-cause mortality in CKD (12).

FGF23 binds to Klotho and FGF receptors to exert its physiological effects on traditional, on-target organs, such as the kidney and parathyroid glands, thereby regulating phosphate homeostasis and mineral metabolism. Recently, it has been shown that FGF23 could also target cell types that lack Klotho. In CKD patients, excess FGF23 also exerts Klotho-independent effects on non-traditional, off-target organs, such as the heart, cells of the immune system, and the liver (14). The off-target effect is activated at high FGF23 concentrations and may cause pathologic cellular changes, leading to poor outcomes in CKD patients (15). Therefore, it is meaningful to examine the effects of the relative ratio of Klotho and FGF23 on the outcome of CKD patients, rather than investigating Klotho and FGF23 individually. High FGF23 is associated with greater risks of severe inflammation (16, 17), and chronic inflammation is involved in renal progression (18). Therefore, FGF23 may affect renal progression in CKD patients. There is little data on the long-term clinical outcomes of Klotho/FGF23 together in CKD patients. Therefore, we aimed to investigate the association between the Klotho/FGF23 ratio and renal progression, all-cause mortality, and CV outcomes in CKD patients including all CKD stages, using data from a large-scale Korean CKD cohort. Changes in CV parameters at the time of enrollment and during follow-up according to the Klotho/FGF23 ratio were also examined.

## Methods

### Study Design and Population

The KoreaN Cohort Study for Outcomes in Patients With Chronic Kidney Disease (KNOW-CKD) was a multicenter prospective cohort study in Korea that enrolled subjects with CKD stages 1 to 5 (predialysis) from nine university-affiliated hospitals. The detailed study methods and design of the KNOW-CKD have been described previously (19). Among the 2,238 participants enrolled in the KNOW-CKD between 2011 and 2016, 2,099 subjects whose serum Klotho and FGF23 levels were obtained at enrollment were included in the analysis. The study protocol was approved in 2011 by the ethical committee of each participating clinical center and by the institutional review boards of Seoul National University Hospital (1104-089-359), Yonsei University Severance Hospital (4-2011-0163), Seoul St. Mary's Hospital (KC11OIMI0441), Seoul National University Bundang Hospital (B-1106/129-008), Kangbuk Samsung Medical Center (2011-01-076), Gil Hospital (GIRBA2553), Eulji General Hospital (201105-01), Chonnam National University Hospital (CNUH-2011-092), and Pusan Paik Hospital (11-091). All study subjects provided written informed consent. The study protocol followed the principles of the Declaration of Helsinki.

### Clinical Data Collection and Laboratory Measurements

Baseline demographic characteristics such as age, sex, body mass index (BMI), comorbidities, cause of CKD, and laboratory parameters at enrollment were extracted from an electronic data management system (http://www.phactax.org) with assistance from the Division of Data Management at the Seoul National University Medical Research Collaborating Center. Patients with a history of diabetes mellitus (DM), a fasting serum glucose ≥126 mg/dL, or those on anti-diabetic medication were considered to have DM. Patients with a history of hypertension (HTN), a systolic blood pressure ≥140 mmHg or diastolic blood pressure ≥90 mmHg, or those on antihypertensive drugs were considered to have HTN. Patients considered to have CV disease were those with a history of coronary artery disease, cerebrovascular disease, congestive heart failure, arrhythmia, or peripheral vascular disease. The following laboratory variables were measured using a ≥8-h fasting blood sample at each participating center: hemoglobin, uric acid, albumin, total cholesterol, C-reactive protein, phosphorus, calcium, and intact parathyroid hormone (PTH). Serum creatinine was measured at a central laboratory (Lab Genomics, Korea) using an isotope dilution mass spectrometry-traceable method (20). The estimated glomerular filtration rate (eGFR) was determined using the Chronic Kidney Disease Epidemiology Collaboration (CKD-EPI) creatinine equation (21). CKD stages were defined according to the Kidney Disease: Improving Global Outcomes guidelines (22). Second voided or random urine samples were immediately sent to a central laboratory to measure urine creatinine and protein levels. The urinary protein excretion was quantified using the random urinary protein-to-creatinine ratio (UPCR, g/g). Alcohol consumption pattern was investigated: non-drinker, occasional drinker (<6 standard drinks/week), regular drinker (≥6 standard drinks/week), moderate drinker (<5 standard drinks/occasion and no alcohol-related problem within the past year), binge drinker [≥5 standard drinks/occasion (23) or the presence of an alcohol-related problem within the past year]. Physical activity was measured using the Korean form of the International Physical Activity Questionnaire (24, 25). Health-enhancing physical activity was defined as achieving at least 150 min/week of moderate-intensity physical activity, 75 min/week of vigorous-intensity physical activity, or an equivalent combination of moderate-vigorous physical activity (MVPA) (26). Frequency of MVPA per week was also investigated.

### Klotho and FGF23 Measurement

The serum α-Klotho level was measured using a commercial enzyme-linked immunosorbent assay (ELISA) kit (Immuno-Biological Laboratories Co., Gunma, Japan) according to the manufacturer's protocol. The intra-assay and inter-assay coefficients of variation were 2.7–3.5% (Klotho levels: 186.64–2,968.78 pg/mL) and 2.9–11.4% (Klotho levels: 165.47–2,903.01 pg/mL), respectively. Serum C-terminal FGF23 was measured using a commercial ELISA kit (Immutopics, San Clemente, CA, USA) according to the manufacturer's protocol. The intra-assay and inter-assay coefficients of variation as reported by the manufacturer were 1.4–2.4% (FGF23 levels: 33.7–302 RU/mL) and 2.4–4.7% (FGF23 levels: 33.6–293 RU/mL), respectively.

### Echocardiographic and Cardiovascular Parameters

Two-dimensional echocardiography was conducted, and left ventricular (LV) mass index was calculated by dividing the LV mass by the body surface area. Left ventricular hypertrophy (LVH) was defined as an LV mass index >115 g/m^3^ in men and >95 g/m^3^ in women, according to the American Society of Echocardiography guidelines (27). LV geometry was classified by LV mass index and relative wall thickness (RWT = [2 × PWTd]/LVIDd) into the following categories: normal geometry (normal LVMI with a RWT ≤ 0.42); concentric remodeling (normal LVMI with a RWT > 0.42); eccentric LVH (LVH with a RWT ≤ 0.42); concentric LVH (LVH with a RWT > 0.42). LV ejection fraction and the ratio (E/E′ ratio) of mitral peak velocity of early filling (E) to the early diastolic mitral annular velocity (E′) were evaluated to find systolic and diastolic dysfunction, respectively. Abdominal aorta calcification (AAC) score (28) and coronary artery calcification score (CACS) (29, 30) were measured to evaluate vascular calcification. The presence of abdominal aortal calcification was defined as a AAC score ≥1 in the present study. The presence of coronary artery calcification was defined as a CACS >100 in the present study (31). The ankle-brachial index was also measured (32).

### Study Outcomes

The primary outcome was renal events, defined as a composite of a 50% decrease in eGFR from baseline, doubling of serum creatinine level, or development of ESKD. ESKD was defined as the initiation of renal replacement therapy, including dialysis or renal transplantation. The secondary composite outcome consisted of CV events and all-cause mortality. Patients were followed until March 2020. The eGFR decline during the follow-up period was also analyzed. In subgroup analyses, changes in echocardiography parameters, CACS, and AAC scores at 4 years of follow-up were investigated.

### Statistical Analyses

Continuous variables were analyzed using analysis of variance or Kruskal–Wallis test. The Kolmogorov–Smirnov test was used to analyze the normality of the distributions of parameters. The results were presented as mean ± standard deviation for variables with normal distributions and as median (interquartile range) for variables with skewed distributions. Categorical variables were evaluated using the χ^2^-test or Fisher's exact test and were presented as frequencies and percentages. The Klotho to FGF23 ratio was calculated as Klotho values divided by FGF23 values + 1 (hereinafter called the Klotho/FGF23 ratio). A log transformation was used to normalize the Klotho/FGF23 ratio. Participants were categorized into quartiles according to Klotho/FGF23 ratio. Cox proportional hazards models with adjustments, including variables that were significant in a univariable analysis or other clinically relevant variables, were used to analyze the association between the Klotho/FGF23 ratios and study outcomes. The results were expressed as hazard ratios (HRs) and 95% confidence intervals (CIs). Subjects who were lost to follow-up were censored at the date of their last examination. The rates of renal function decline per year were determined using the slope of eGFR analyzed using a generalized linear mixed model. Only 1,851 (88.1%) patients whose eGFR values were measured three or more times during the follow-up period were included in the eGFR decline analysis. The rapid decline of eGFR was defined as eGFR <-3 ml/min/1.73 m^2^/year. Binary logistic regression analysis was used to identify the risk factors for the rapid decline of kidney function. Multivariable linear regression model analysis was also used to investigate the association between eGFR slope and Klotho/FGF23 ratio. In addition, Harrell's C-index and receiver operating characteristic (ROC) curve analysis were conducted to evaluate the prognostic value of Klotho and FGF23 levels and the Klotho/FGF23 ratio for renal events. *P-*values < 0.05 were considered statistically significant. The SPSS statistical software (SPSS version 20.0, IBM Corporation, Armonk, NY, USA) was used for all analyses.

## Results

### Baseline Clinical Characteristics of the Study Subjects

The clinical characteristic of the study subjects by Klotho/FGF23 ratio quartiles are shown in Table 1. The mean age was 53.6 ± 12.2 years, and 1,280 (61.0%) of the subjects were male. The mean eGFR was 53.0 ± 30.7 mL/min/1.73 m^2^. The mean age was younger in the high Klotho/FGF23 ratio quartiles (*P* = 0.020). The prevalence of DM (*P* < 0.001), HTN (*P* < 0.001), and CV disease (*P* = 0.008) was lower in the high Klotho/FGF23 ratio quartiles. Estimated GFR (*P* < 0.001) and hemoglobin (*P* < 0.001) were higher in the high Klotho/FGF23 ratio quartiles. Serum phosphorus (*P* < 0.001) and PTH (*P* < 0.001) were lower in the high Klotho/FGF23 ratio quartiles. Binge drinkers were more in the 4th Klotho/FGF23 ratio group (*P* = 0.033). Health-enhancing physical activity was lower in the 1st and 4th Klotho/FGF23 ratio groups (*P* = 0.022).

**Table 1:** Clinical characteristics of the study subjects at enrollment, stratified by Klotho/FGF23 ratio quartiles.

		**Klotho/FGF23 ratio**				
**Characteristics**	**Total (*N* = 2,099)**	**1st quartile (*n* = 524)**	**2nd quartile (*n* = 525)**	**3rd quartile (*n* = 525)**	**4th quartile (*n* = 525)**	***P*-value**
Age (mean ± SD)	53.6 ± 12.2	54.4 ± 12.0	54.4 ± 12.2	52.7 ± 12.0	52.8 ± 12.6	0.020
Sex, male, *n* (%)	1,280 (61.0)	304 (58.0)	342 (65.1)	315 (60.0)	319 (60.8)	0.113
BMI (kg/m^2^)	24.6 ± 3.4	24.5 ± 3.5	24.8 ± 3.5	24.6 ± 3.3	24.4 ± 3.2	0.374
SBP (mmHg)	127.8 ± 16.2	131.4 ± 19.0	126.7 ± 15.0	126.6 ± 15.1	126.4 ± 14.7	<0.001
DM, *n* (%)	711 (33.9)	228 (43.5)	188 (35.9)	155 (29.5)	140 (26.8)	<0.001
HTN, *n* (%)	2,013 (95.9)	510 (97.3)	508 (96.8)	506 (96.4)	489 (93.1)	0.003
Preexisting CV disease, *n* (%)	334 (15.9)	104 (19.8)	90 (17.1)	70 (13.3)	70 (13.3)	0.008
CAD, *n* (%)	129 (6.1)	42 (8.0)	38 (7.2)	24 (4.6)	25 (4.8)	0.077
Cerebrovascular ds, *n* (%)	131 (6.2)	38 (7.8)	35 (6.7)	31 (5.9)	27 (5.1)	0.520
HF, *n* (%)	30 (1.4)	12 (2.3)	4 (0.8)	7 (1.3)	7 (1.3)	0.214
Arrhythmia, *n* (%)	54 (2.6)	22 (4.2)	13 (2.5)	11 (2.1)	8 (1.5)	0.068
PVD, *n* (%)	77 (3.7)	24 (4.6)	24 (4.6)	14 (2.7)	15 (2.9)	0.178
Cause of CKD						<0.001
DN, *n* (%)	490 (23.3)	171 (32.6)	131 (25.0)	109 (20.8)	79 (15.0)	
Hypertension, *n* (%)	388 (18.5)	84 (16.0)	101 (19.2)	92 (17.5)	111 (21.1)	
GN, *n* (%)	746 (35.5)	155 (29.6)	183 (34.9)	202 (38.5)	206 (39.2)	
PKD, *n* (%)	346 (16.5)	81 (15.5)	80 (15.2)	91 (17.3)	94 (17.9)	
Others, *n* (%)	129 (6.1)	33 (6.3)	30 (5.7)	31 (5.9)	35 (6.7)	
Smoking status, *n* (%)						0.022
Never	1,117 (53.3)	269 (51.3)	256 (49.0)	304 (57.9)	288 (54.9)	
Current or former	979 (46.7)	255 (48.7)	266 (51.0)	221 (42.1)	237 (45.1)	
Klotho (Q1, Q3) (pg/mL)	536 (419, 666)	444 (335, 565)	547 (451, 644)	591 (462, 739)	556 (429, 718)	<0.001
FGF23 (Q1, Q3) (RU/mL)	19.6 (1.7, 34.6)	51.6 (36.3, 76.7)	26.2 (21.2, 32.5)	9.7 (4.8, 17.8)	0.04 (0.0, 0.5)	<0.001
eGFR (mL/min/1.73 m^2^)	53.0 ± 30.7	41.2 ± 29.4	48.5 ± 26.9	59.5 ± 30.2	62.8 ± 31.3	<0.001
Hemoglobin (g/dL)	12.8 ± 2.0	11.9 ± 2.0	12.9 ± 2.0	13.2 ± 1.9	13.4 ± 1.8	<0.001
Uric acid (mg/dL)	7.0 ± 1.9	7.4 ± 2.0	7.2 ± 1.8	6.9 ± 1.9	6.7 ± 1.9	<0.001
Albumin (g/dL)	4.2 ± 0.4	4.1 ± 0.5	4.2 ± 0.4	4.2 ± 0.4	4.2 ± 0.4	<0.001
Total cholesterol (mg/dL)	174.3 ± 39.4	172.8 ± 42.4	172.2 ± 38.2	175.6 ± 36.5	176.6 ± 38.2	0.199
CRP, median, (Q1, Q3) (mg/L)	0.6 (0.2, 1.7)	0.7 (0.3, 2.0)	0.7 (0.3, 1.6)	0.5 (0.2, 1.4)	0.7 (0.2, 1.5)	0.018
Phosphorus (mg/dL)	3.7 ± 0.7	4.0 ± 0.8	3.7 ± 0.6	3.6 ± 0.6	3.5 ± 0.6	<0.001
Corrected Ca (mg/dL)^*^	9.0 ± 0.4	8.9 ± 0.5	9.0 ± 0.5	9.0 ± 0.4	9.0 ± 0.4	<0.001
PTH, median (Q1, Q3) (pg/mL)	51.5 (33.3, 84.0)	70.9 (41.3, 122.5)	53.1 (36.0, 81.7)	45.5 (31.0, 74.0)	44.7 (29.1, 69.1)	<0.001
UPCR (Q1, Q3) (g/g)	0.49 (0.14, 1.51)	0.73 (0.24, 2.30)	0.57 (0.17, 1.66)	0.40 (0.12, 1.12)	0.33 (0.09, 0.97)	<0.001
Urine calcium (mg/day)	45 (21.6, 95.8)	33.0 (16.2, 69.4)	39.1 (19.5, 84.2)	50.0 (23.0, 110.5)	65.0 (29.6, 122.6)	<0.001
Urine phosphorus (mg/day)	571 (400, 737)	500 (400, 700)	588 (400, 770)	600 (400, 780)	600 (400, 793)	0.036
Medications						
ACEi or ARB, *n* (%)	1,797 (85.7)	450 (85.9)	449 (85.9)	464 (88.4)	434 (82.7)	0.071
Diuretics, *n* (%)	670 (32.0)	225 (42.9)	168 (32.1)	129 (24.6)	148 (28.2)	<0.001
Ca-based phosphorus binder, *n* (%)	183 (8.7)	69 (13.2)	39 (7.5)	40 (7.6)	35 (6.7)	0.001
Active vitamin D, *n* (%)	50 (2.4)	21 (4.0)	9 (1.7)	10 (1.9)	10 (1.9)	0.047
LV mass index (g/m^3^)	93.1 ± 24.5	99.9 ± 28.7	92.8 ± 22.8	90.7 ± 22.0	89.2 ± 22.4	<0.001
LVH, *n* (%)^**^	512 (25.0)	179 (35.1)	123 (24.0)	103 (20.2)	107 (20.7)	<0.001
LV ejection fraction (%)	64.0 ± 6.3	64.1 ± 7.1	63.9 ± 5.9	64.3 ± 6.1	63.8 ± 6.1	0.542
E/E′	9.9 ± 3.9	10.6 ± 4.9	10.0 ± 3.4	9.7 ± 3.4	9.4 ± 3.6	<0.001
AAC ≥ 1, *n* (%)	704 (35.1)	227 (45.4)	175 (35.3)	160 (32.1)	142 (27.9)	<0.001
CACS (Q1, Q3)	1 (0, 82)	5 (0, 140)	1 (0, 94)	0 (0, 62)	0 (0, 42)	<0.001
CACS >100, *n* (%)	454 (23.0)	133 (27.5)	123 (24.7)	107 (21.4)	91 (18.5)	0.005
Ankle-brachial index	1.1 ± 0.1	1.2 ± 0.1	1.1 ± 0.1	1.1 ± 0.1	1.1 ± 0.1	0.080
Binge drinker^†^, *n* (%)	318 (16.7)	80 (16.8)	73 (15.4)	75 (15.89)	90 (18.9)	0.033
Health-enhancing physical activity^††^, *n* (%)	774 (42.4)	170 (37.6)	209 (45.4)	212 (46.2)	183 (40.1)	0.022
Frequency of MVPA per week (Q1, Q3)	1 (0, 4)	0 (0, 4)	1 (0, 5)	1 (0, 4)	0 (0, 4.7)	0.034

* *Corrected Ca (mg/dL) = measured total Ca (mg/dL) + 0.8 × [4 – measured serum albumin (g/dL)]*.

** *LVH was defined as LV mass index > 115 g/m^3^ in men and > 95 g/m^3^ in women*.

† *Binge drinker was defined as the consumption of ≥ 5 standard drinks per occasion or the presence of an alcohol-related problem within the past year*.

†† *Health-enhancing physical activity was defined as achieving at least 150 min/week of moderate-intensity physical activity, 75 min/week of vigorous-intensity physical activity, or an equivalent combination of MVPA*.

*FGF23, fibroblast growth factor 23; SD, standard deviation; BMI, body mass index; SBP, systolic blood pressure; DM, diabetes mellitus; HTN, hypertension; CV, cardiovascular; CAD, coronary artery disease; HF, heart failure; PVD, peripheral vascular disease; CKD, chronic kidney disease; DN, diabetic nephropathy; GN, glomerulonephritis; PKD, polycystic kidney disease; eGFR, estimated glomerular filtration rate as determined by the CKD-EPI creatinine equation; CRP, C-reactive protein; Ca, calcium; PTH, parathyroid hormone; UPCR, urine protein creatinine ratio; ACEi, angiotensin converting enzyme inhibitor; ARB, angiotensin II receptor blocker; LV, left ventricular; LVH, left ventricular hypertrophy; EF, ejection fraction; E/E′, ratio of mitral peak velocity of early filling (E) to early diastolic mitral annular velocity (E′); AAC, Abdominal aorta calcification; CACS, coronary artery calcium score; MVPA, Moderate-vigorous physical activity*.

### Klotho/FGR23 Ratio and Renal Events

**Figure 1:**
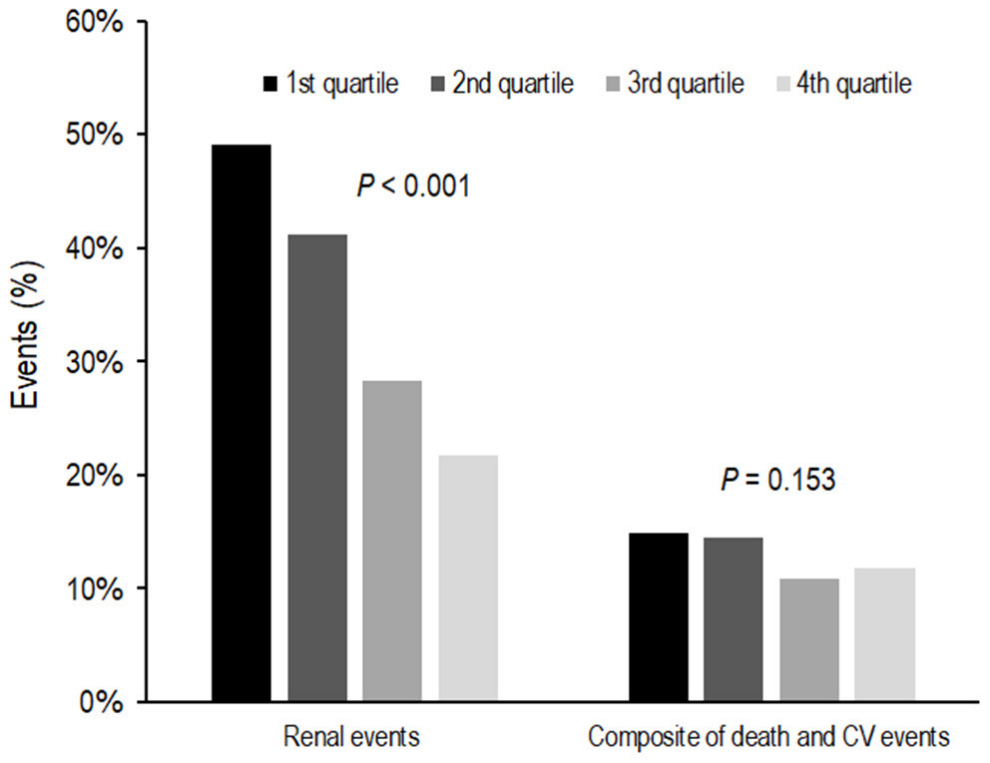
Event rates for renal events and composites of death and CV events according to Klotho/FGF23 ratio. The first quartile of the Klotho/FGF23 ratio group was at a greater risk of developing renal events compared to the other quartile groups (*P* < 0.001). Composites of death and CV events were not significantly different according to Klotho/FGF23 ratio groups (*P* = 0.153). FGF23, fibroblast growth factor 23; CV, cardiovascular.

**Figure 2:**
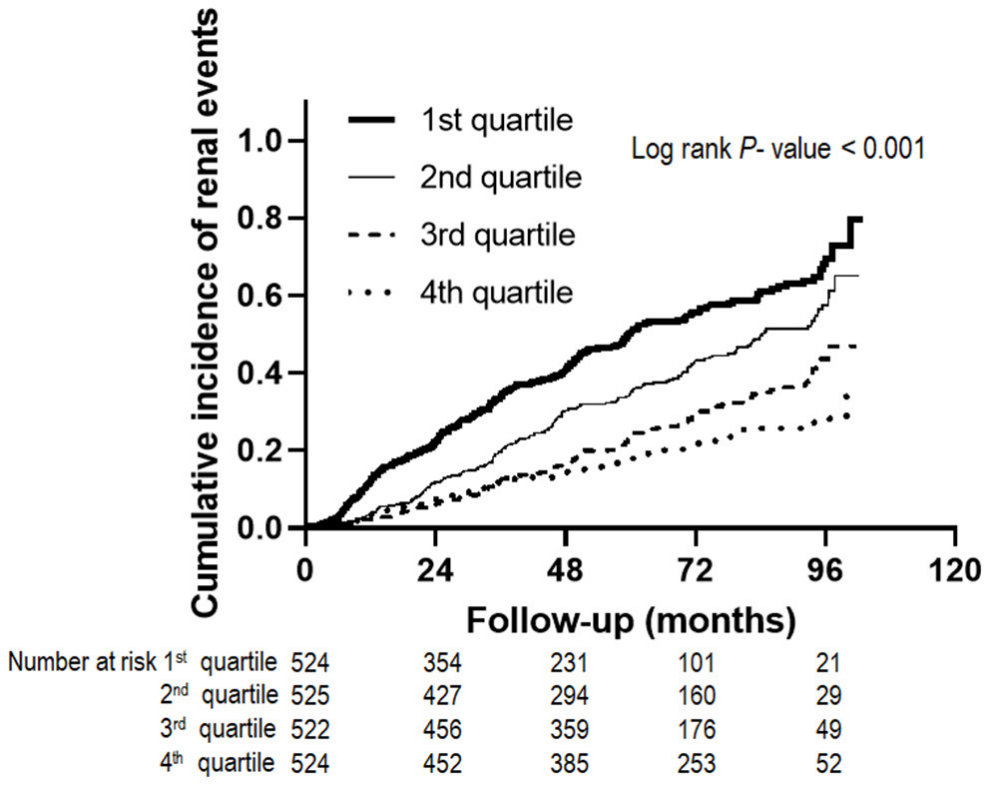
Renal events according to Klotho/FGF23 ratio. The Kaplan-Meier curves show that the low Klotho/FGF23 ratio group had a significantly higher cumulative incidence of renal events (*P* < 0.001). FGF23, fibroblast growth factor 23.

During the follow-up period of 64.0 ± 28.2 months, 735 (35.1%) subjects developed renal events. Figure 1 presents the renal events according to the Klotho/FGF23 ratio groups. The first quartile of Klotho/FGF23 ratio group was at a greater risk of developing renal events compared to the other quartile groups (*P* < 0.001). The Kaplan–Meier curves showed that the low Klotho/FGF23 ratio group had a significantly higher cumulative incidence of renal events (*P* < 0.001; Figure 2). The multivariable Cox regression analysis presented that the first (HR: 1.36; 95% CI: 1.08–1.72, *P* = 0.010) and second (HR: 1.45; 95% CI: 1.15–1.83, *P* = 0.002) quartiles of the Klotho/FGF23 ratio group showed increased renal events as compared to the fourth quartile group (Table 2). As a continuous variable, as the log (Klotho/FGF23 ratio) increased, the development of renal events decreased (HR: 0.85; 95% CI: 0.75–0.96, *P* = 0.008). Similarly, when physical activity, smoking status, and alcohol consumption variables were added in model 4, the low Klotho/FGF23 ratio was significantly associated with developing renal events. To confirm whether the Klotho/FGF23 ratio is a valuable predictor of renal events, we compared Harrell's C-index between Klotho and FGF23 levels and the Klotho/FGF23 ratio described in the statistical analysis section. The Harrell's C-index for Klotho/FGF23 ratio was 0.644. The Harrell's C-indices for Klotho and FGF23 were 0.535 and 0.642, respectively. Furthermore, Harrell's C-index for Klotho/FGF23 ratio added to an adjusted model (model 4) was 0.841. When Klotho and FGF23 levels were added instead of Klotho/FGF23 ratio in the adjusted model (model 4), Harrell's C-index was 0.840. These findings suggest that Klotho/FGF23 ratio can have predictive value for renal events development.

**Table 2:** Renal events according to Klotho/FGF23 ratio.

	**Model 1**		**Model 2**		**Model 3**		**Model 4**	

**Klotho/FGF23 ratio**	**HR (95% CI)**	***P*** **-value**	**HR (95% CI)**	***P*** **-value**	**HR (95% CI)**	***P*** **-value**	**HR (95% CI)**	***P*** **-value**
**Categorical variable**
1st quartile	3.52 (2.82, 4.39)	<0.001	2.92 (2.33, 3.65)	<0.001	1.37 (1.08, 1.73)	0.008	1.36 (1.08, 1.72)	0.010
2nd quartile	2.31 (1.84, 2.90)	<0.001	2.12 (1.69, 2.66)	<0.001	1.44 (1.14, 1.81)	0.002	1.45 (1.15, 1.83)	0.002
3rd quartile	1.43 (1.12, 1.83)	0.004	1.35 (1.05, 1.72)	0.017	1.25 (0.98, 1.60)	0.079	1.26 (0.98, 1.62)	0.068
4th quartile	Reference	-	Reference	-	Reference	-	Reference	-
**Continuous variable**
Klotho/FGF23 ratio^*^	0.47 (0.42, 0.53)	<0.001	0.52 (0.46, 0.58)	<0.001	0.84 (0.75, 0.95)	0.007	0.85 (0.75, 0.96)	0.008

*Model 1: Unadjusted*.

*Model 2: Adjusted for age, sex, HTN, DM, preexisting CVD, systolic blood pressure, BMI*.

*Model 3: Model 2 + eGFR, phosphorous, corrected calcium, hemoglobin, log PTH*.

*Model 4: Model 3 + ACEi or ARB use, diuretics, Ca-baseline phosphorus binder, and active vitamin D use*.

*FGF23, fibroblast growth factor 23; HR, hazard ratio; CI, confidence interval; DM, diabetes mellitus; HTN, hypertension; CVD, cardiovascular disease; BMI, body mass index; eGFR, estimated glomerular filtration rate by CKD-EPI creatinine equation; PTH, parathyroid hormone; ACEi, angiotensin converting enzyme inhibitor; ARB, angiotensin II receptor blocker; Ca, calcium*.

* *Data for Klotho/FGF23 ratio was log transformed*.

### Klotho/FGR23 Ratio and CV Events and All-Cause Mortality

During the follow-up period, 273 subjects developed a composite of CV events and death. Composites of death and CV events were not significantly different among the Klotho/FGF23 ratio groups (*P* = 0.153; Figure 1). In an unadjusted Cox proportional hazards model, the logarithm of the Klotho/FGF23 ratio was inversely associated with the composite of CV events and death (HR: 0.72; 95% CI: 0.60–0.87, *P* < 0.001; Table 3). After adjustment, there was no significant association between the Klotho/FGF23 ratio groups and the composite outcome of CV events and death (Table 3).

**Table 3:** Composite of mortality and cardiovascular events according to Klotho/FGF23 ratio.

	**Model 1**		**Model 2**		**Model 3**		**Model 4**	

**Klotho/FGF23 ratio**	**HR (95% CI)**	**P-value**	**HR (95% CI)**	**P-value**	**HR (95% CI)**	**P-value**	**HR (95% CI)**	**P-value**
**Categorical variable**
1st quartile	1.44 (1.03, 2.01)	0.034	1.07 (0.76, 1.51)	0.691	0.92 (0.64, 1.33)	0.668	0.93 (0.65, 1.34)	0.694
2nd quartile	1.34 (0.96, 1.88)	0.085	1.09 (0.78, 1.53)	0.613	1.01 (0.71, 1.42)	0.972	1.03 (0.73, 1.46)	0.879
3rd quartile	0.98 (0.69, 1.41)	0.932	0.91 (0.64, 1.31)	0.619	0.89 (0.62, 1.28)	0.525	0.91 (0.63, 1.32)	0.629
4th quartile	Reference	-	Reference	-	Reference	-	Reference	-
**Continuous variable**
Klotho/FGF23 ratio^*^	0.72 (0.60, 0.87)	<0.001	0.86 (0.72, 1.04)	0.117	0.95 (0.78, 1.15)	0.614	0.95 (0.78, 1.15)	0.605

*Model 1: Unadjusted*.

*Model 2: Adjusted for age, sex, HTN, DM, preexisting CVD, systolic blood pressure, BMI*.

*Model 3: Model 2 + eGFR, phosphorous, corrected calcium, hemoglobin, log PTH*.

*Model 4: Model 3 + ACEi or ARB use, diuretics, Ca-baseline phosphorus binder, and active vitamin D use*.

*FGF23, fibroblast growth factor 23; HR, hazard ratio; CI, confidence interval; DM, diabetes mellitus; HTN, hypertension; CVD, cardiovascular disease; BMI, body mass index; eGFR, estimated glomerular filtration rate by CKD-EPI creatinine equation; PTH, parathyroid hormone; ACEi, angiotensin converting enzyme inhibitor; ARB, angiotensin II receptor blocker; Ca, calcium*.

* *Data for Klotho/FGF23 ratio was log transformed*.

### Association of Klotho/FGR23 Ratio With Renal Function Decline

**Figure 3:**
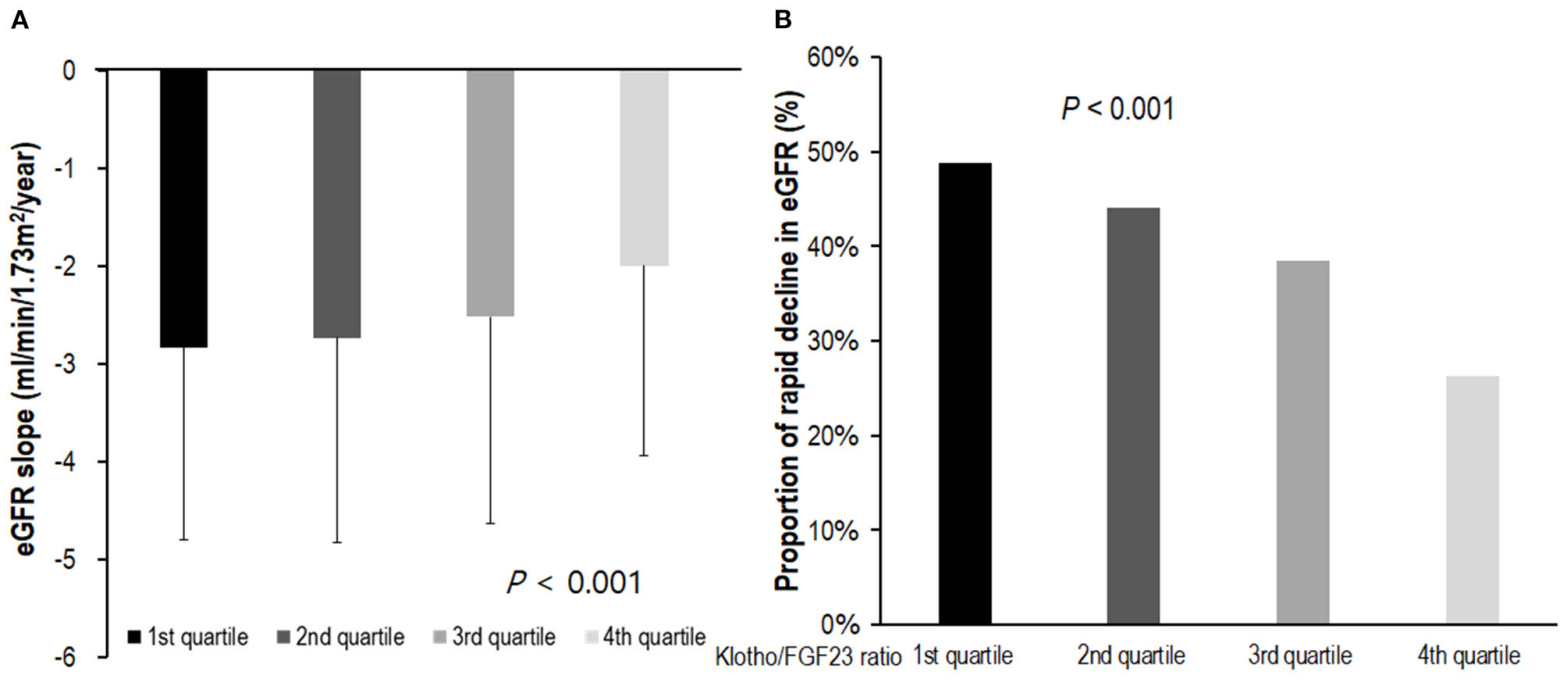
The eGFR slope and rapid decline of eGFR according to Klotho/FGF23 ratio. **(A)** The eGFR slope according to Klotho/FGF23 ratio; **(B)** rapid decline of eGFR according to Klotho/FGF23 ratio. The eGFR slope was analyzed in 1,851 patients for whom eGFR was measured more than three times during the follow-up period. The eGFR slope was lower in the low Klotho/FGF23 ratio group (*P* < 0.001; **A**). The rapid decline of kidney function was defined as an estimated glomerular filtration rate (eGFR) <-3 ml/min/1.73 m^2^/year. The proportion of patients exhibiting a rapid decline of eGFR was higher in the first quartile of Klotho/FGF23 ratio group (*P* < 0.001; **B**). eGFR, estimated glomerular filtration rate by CKD-EPI creatinine equation; FGF23, fibroblast growth factor 23.

We analyzed renal function decline as the slope of eGFR for 1,851 patients whose eGFR values were measured three times or more during the follow-up period. The eGFR slope was lower in the low Klotho/FGF23 ratio group (*P* < 0.001; Figure 3A). The proportion of patients showing a rapid decline of eGFR was higher in the first quartile of Klotho/FGF23 ratio group (*P* < 0.001; Figure 3B). The multivariable binary logistic regression analysis revealed that the first [odds ratio (OR): 1.68; 95% CI: 1.23–2.28, *P* = 0.001], second (OR: 1.73; 95% CI: 1.29–2.32, *P* < 0.001), and third (OR: 1.63; 95% CI: 1.21–2.19, *P* = 0.001) quartiles of Klotho/FGF23 ratio groups showed a significantly rapid decline in eGFR compared to the fourth quartile group. eGFR slope was significantly associated with log transformed Klotho/FGF23 ratio (β: 0.26; 95% CI: 0.12–0.41; *P* < 0.001; Supplementary Table S1).

### Echocardiography and Vascular Calcification Parameters

We further examined the relationship between the Klotho/FGF2 ratio and CV parameters. Baseline echocardiography and vascular calcification parameters are shown in Table 1. LV mass index (*P* < 0.001) and E/E′ (*P* < 0.001) were higher in the lower Klotho/FGF23 ratio groups. The LV geometry pattern according to Klotho/FGF23 ratio is shown in Supplementary Figure S1. Low Klotho/FGF23 ratio quartiles showed greater increases in the prevalence of LVH (Supplementary Figure S1A). AAC score ≥1 was 45.4% in the first Klotho/FGF23 ratio group and higher in the lower quartiles (*P* < 0.001). CACS > 100 was 27.5% in the first Klotho/FGF23 ratio group and was also higher in the lower quartiles (*P* = 0.005). Ankle-brachial index was similar according to Klotho/FGF23 ratio quartiles.

At 4 years of follow-up, echocardiography and vascular calcification parameters were evaluated in approximately 59% of subjects. Fourth-year echocardiography and vascular calcification parameters are presented in Supplementary Table S2. LV mass index (*P* = 0.012) and E/E′ (*P* = 0.001) at 4 years were higher in the low Klotho/FGF23 ratio quartiles. The prevalence of LVH was higher in the first Klotho/FGF23 ratio quartile at 4 years (Supplementary Figure S1B). Of the 987 patients who did not have LVH at baseline, 135 (13.7%) subjects developed *de novo* LVH. *De novo* LVH incidence was higher in the first Klotho/FGF23 ratio quartile (first, second, third, and fourth quartiles: 20.3, 14.7, 11.3, and 11.1%, respectively; *P* = 0.020). The presence of abdominal aortal calcification (*P* = 0.001) and coronary artery calcification (*P* = 0.038) were also higher in the low Klotho/FGF23 ratio quartiles.

## Discussion

In the present study, the incidence of renal events during the follow-up period was higher in low Klotho/FGF23 ratio quartile. Subjects in the first quartile Klotho/FGF23 ratio group showed a significantly rapid decline in eGFR. In our study, there was no significant association between the Klotho/FGF23 ratio and the composite outcome of CV events and death. The presence of LVH and vascular calcification were higher in the low Klotho/FGF23 ratio quartile group at enrollment. In subgroup analysis, during the follow-up period, LV mass index and development of *de novo* LVH were higher in the low Klotho/FGF23 ratio quartile group. The presence of vascular calcification was also higher in the low Klotho/FGF23 ratio quartiles.

A previous study reported that patients with higher soluble Klotho levels exhibited a reduced risk of adverse kidney outcomes, including ESKD, and of serum creatinine doubling in CKD stage 3–5 (33). In this study, FGF23 was not a significant risk factor for renal events after variable adjustment. Another study showed that low Klotho and high FGF23 were significant risk factors of composite renal outcomes including serum creatinine doubling, ESKD, and death (8). In this study, the areas under the ROC curve for soluble Klotho and FGF23 were comparable. Intact serum FGF23 was a predictor of doubling of creatinine, dialysis initiation, and death in diabetic nephropathy patients (34). In another study, FGF23 was a risk factor for dialysis initiation alone or dialysis initiation and death in advanced CKD (eGFR <30 m/min/1.73 m^2^) (35). Klotho and FGF23 are earlier markers of CKD-MBD that change before the alteration of such biochemical parameters as phosphorus and PTH, and the Klotho/FGF23 axis could be an early marker for the outcome of CKD patients. Furthermore, Klotho acts like a hormone that exerts anti-senescent, anti-oxidant, and anti-apoptotic effects (36, 37). In previous experimental studies, Klotho was reduced in kidney injury, and kidney function and tubulointerstitial injury improved when the Klotho gene was transferred to the damaged kidney (38–40).

Although FGF23 has Klotho-dependent traditional and on-target effects, a recent study showed that FGF23 also had Klotho-independent, non-traditional, off-target effects (14, 15). Pathologically increased FGF23 causes hypertrophy in heart cardiomyocytes (41–43) and inflammation in liver hepatocytes (44). Off-target effects of FGF23 also affect immune cells such as neutrophils and macrophages. A previous *in vitro* study showed that FGF23 was released by proinflammatory M1 macrophages and acted locally to increase tumor necrosis factor-α (TNF-α) production in M0 macrophages in the absence of Klotho (45). In animal experiments using a murine CKD model, FGF23 regulated genes involved in inflammation and renal fibrosis (transforming growth factor-β, TNF-α) (46). In addition, FGF23 inhibited neutrophil recruitment in a Klotho-independent manner in CKD (47). CKD is a state of acquired immune deficiency involving humoral and cellular immunity (48). As renal progression in CKD is associated with macrophage tissue infiltration and inflammation, these off-target effects of FGF23 related to the immune system provide a possible mechanistic link between elevated FGF23 and renal progression (14). Because FGF23 acts on target tissues with and without Klotho, it is crucial to evaluate their effects in CKD patients, not only for Klotho or FGF23 alone, but also the relative ratio of Klotho and FGF23. In the present study, a low Klotho/FGF23 ratio was associated with renal events after adjustment. In addition, the area under the ROC of the Klotho/FGF23 ratio was higher than those of either Klotho and FGF23 alone.

In the present study, a low Klotho/FGF23 ratio was associated with the presence of LVH and vascular calcification. At baseline, the lower Klotho/FGF23 ratio quartile group was associated with the presence of LVH and vascular calcification. In addition, after follow-up, the lower Klotho/FGF23 ratio quartile group exhibited a higher incidence of *de novo* development of LVH and vascular calcification. Of the 987 patients who did not have LVH at baseline, 135 (13.7%) subjects developed *de novo* LVH, and it developed more frequently in the low Klotho/FGF23 ratio group. In an experimental study, Klotho-deficient CKD mice had accelerated cardiac hypertrophy and cardiac fibrosis compared to wild-type CKD mice (49). Intravenous delivery of a transgene encoding soluble Klotho mitigated cardiac hypertrophy in the Klotho-deficient CKD mice. Serum Klotho levels are related with the development of LVH in CKD patients (50). Faul et al. (41) showed that FGF23 can cause LVH independently of Klotho. This indicates that the off-target effects of FGF23, independent of Klotho, affect cardiac myocytes. In patients with elevated FGF23, there have been studies in which aortic or coronary artery calcification scores were higher than in those with lower FGF23 (13), but there have been conflicting results (51). Moreover, up-regulation of Klotho expression protects against vascular calcification in CKD (52). The disruption of the balance between FGF23 and Klotho may be important in vascular calcification, rather than FGF23 or Klotho alone. In our study, the Klotho/FGF23 ratio was not associated with mortality or CV outcome. In the present study, we included all stages of CKD, and patients with early CKD (11.9% with CKD stage 1 and 18.4% with CKD stage 2) were also included. The incidence rates of death and CV outcomes were lower than in another study (35); hence, the results may differ. In addition, statistical significance may be decreased due to the low incidence of death and CV outcomes. However, *de novo* LVH and vascular calcification, which were surrogate parameters of CV outcome, were significantly higher in the low Klotho/FGF23 ratio quartiles.

The advantage of our study is that long-term follow-up results were obtained in a large-scale CKD cohort. Instead of analyzing Klotho or FGF23 alone, we considered the relative ratio of Klotho and FGF23, which might have an interconnection. However, this study also has several limitations. First, Klotho and FGF23 have circadian variations (53), but blood sampling time could not be fixed. Second, we measured C-terminal FGF23 in the present study. Lack of agreement between intact FGF23 and C-terminal FGF23 measurements and also differences in their associations with other biochemical parameters have been reported (54). However, the C-terminal ELISA kit theoretically detects both intact FGF23 and its C-terminal fragments; since virtually all circulating FGF23 is intact, the C-terminal assay measures biologically active FGF23 (55). Third, we did not investigate the phosphorus intake of subjects.

## Conclusion

Low Klotho/FGF23 ratio was significantly associated with increased risk of renal events in this cohort of Korean predialysis CKD patients.

## Data Availability Statement

The original contributions presented in the study are included in the article/Supplementary Material, further inquiries can be directed to the corresponding author.

## Ethics Statement

The studies involving human participants were reviewed and approved by the Ethical Committee of each participating clinical center and the Institutional Review Boards of Seoul National University Hospital (1104-089-359), Seoul National University Bundang Hospital (B-1106/129-008), Yonsei University Severance Hospital (4-2011-0163), Kangbuk Samsung Medical Center (2011-01-076), Seoul St. Mary's Hospital (KC11OIMI0441), Gil Hospital (GIRBA2553), Eulji General Hospital (201105-01), Chonnam National University Hospital (CNUH-2011-092), and Pusan Paik Hospital (11-091). The patients/participants provided their written informed consent to participate in this study.

## Author Contributions

HJK and K-HO were involved with the conception and design of the study. HJK, YK, MK, SK, SKP, SS, YYH, JYJ, CA, and K-HO were involved with patient data collection and acquisition. HJK, MK, SKP, and K-HO performed the analysis and interpretation of data. Article draft and revision were carried out by HJK and K-HO. All authors approved the final manuscript.

## Funding

This study was supported by the Research Program funded by the Korea Center for Disease Control and Prevention (2011E3300300, 2012E3301100, 2013E3301600, 2013E3301601, 2013E3301602, 2016E3300200, 2016E3300201, 2016E3300202, 2019E320100, 2019E320101, and 2022-11-007), the Bio and Medical Technology Development Program of the National Research Foundation (NRF), and funded by the Korean Government (MSIT) (2017M3A9E4044649). The funders had no role in the study design, data collection or analysis, decision to publish, or preparation of the manuscript.

## Conflict of Interest

The authors declare that the research was conducted in the absence of any commercial or financial relationships that could be construed as a potential conflict of interest.

## Publisher's Note

All claims expressed in this article are solely those of the authors and do not necessarily represent those of their affiliated organizations, or those of the publisher, the editors and the reviewers. Any product that may be evaluated in this article, or claim that may be made by its manufacturer, is not guaranteed or endorsed by the publisher.
