# Supplementary material for: Low Klotho/Fibroblast Growth Factor 23 Ratio Is an Independent Risk Factor for Renal Progression in Chronic Kidney Disease: Finding From KNOW-CKD
Source: Front Med (Lausanne). 2022 Jul 8;9:904963. doi: 10.3389/fmed.2022.904963 (PMC9304693; doi:10.3389/fmed.2022.904963)
Supplement: Supplementary file 1 [file Data_Sheet_1.docx]

Supplementary Material

**Table S1. Association between Klotho/FGF23 ratio and eGFR slope**

|  | Model 1 |  | Model 2 |  | Model 3 |  | Model 4 |  |
| --- | --- | --- | --- | --- | --- | --- | --- | --- |
|  | **β (95% CI)** | ***P* value** | **β (95% CI)** | ***P* value** | **β (95% CI)** | ***P* value** | **β (95% CI)** | ***P* value** |
| Klotho/FGF23 ratio^*^ | 0.46  (0.32, 0.60) | <0.001 | 0.43  (0.30, 0.57) | <0.001 | 0.27  (0.12, 0.41) | <0.001 | 0.26  (0.12, 0.41) | <0.001 |

Model 1: Unadjusted

Model 2: Adjusted for age, sex, HTN, DM, preexisting CVD, systolic blood pressure, BMI

Model 3: Model 2 + eGFR, phosphorous, corrected calcium, hemoglobin, log PTH

Model 4: Model 3 + ACEi or ARB use, diuretics, Ca-baseline phosphorus binder, and active vitamin D use

FGF23: fibroblast growth factor 23; eGFR: estimated glomerular filtration rate as determined by the CKD-EPI creatinine equation

^*^Data for Klotho/FGF23 ratio was log transformed.

**Figure S1. Left ventricular geometry pattern at baseline and 4 year**


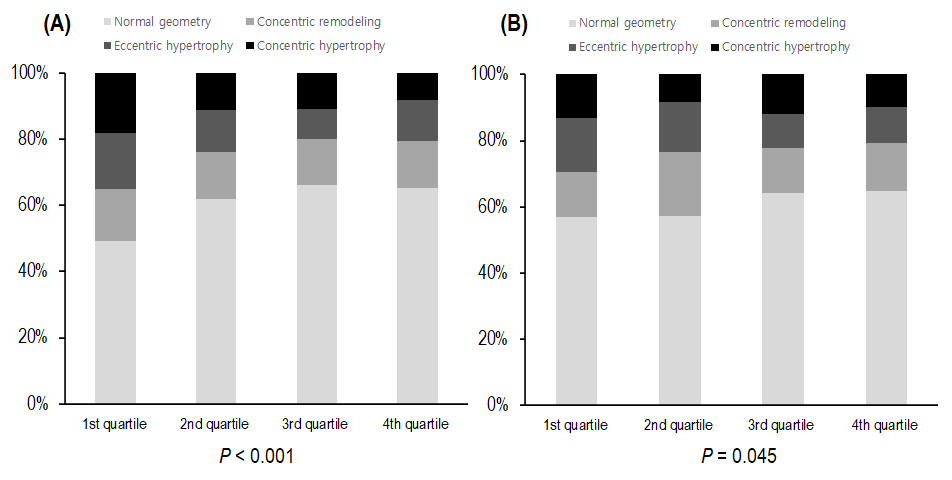


**(A)** Baseline LV geometry pattern, **(B)** 4 year geometry pattern

At 4 years of follow-up, LV geometry pattern was evaluated in approximately 59% of subjects.

LV: left ventricular

**Table S2. Echocardiography and vascular calcification parameters at 4 year**

| Characteristics | Patient number | Total | Klotho/FGF23 ratio | | | | *P*-value |
| --- | --- | --- | --- | --- | --- | --- | --- |
|  |  |  | 1^st^ quartile | 2^nd^ quartile | 3^rd^ quartile | 4^th^ quartile |  |
| LV mass index (g/m^3^) | 1251 | 91.1 ± 22.8 | 94.1 ± 21.3 | 92.9 ± 24.0 | 90.1 ± 22.9 | 88.6 ± 22.4 | 0.012 |
| LVH, n (%)^*^ | 1251 | 292 (23.3) | 69 (29.4) | 69 (23.3) | 78 (22.1) | 76 (20.7) | 0.090 |
| LV ejection fraction (%) | 1256 | 63.9 ± 5.8 | 63.3 ± 6.4 | 64.0 ± 5.7 | 64.0 ± 5.7 | 64.1 ± 5.7 | 0.304 |
| E/E’ | 1250 | 9.8 ± 3.8 | 10.2 ± 4.4 | 10.3 ± 4.2 | 9.6 ± 3.3 | 9.3 ± 3.4 | 0.001 |
| AAC ≥1, n (%) | 1227 | 534 (43.5) | 117 (50.4) | 139 (48.8) | 147 (42.7) | 131 (35.8) | 0.001 |
| CACS >100, n (%) | 1136 | 294 (25.9) | 64 (31.1) | 75 (28.3) | 85 (26.0) | 70 (20.7) | 0.038 |

^*^LVH was defined as LV mass index >115 g/m^3^ in men and > 95 g/m^3^ in women.

LV: left ventricular; LVH: left ventricular hypertrophy; EF: ejection fraction; E/E’: ratio of mitral peak velocity of early filling (E) to early diastolic mitral annular velocity (E’); AAC: Abdominal aorta calcification; CACS: coronary artery calcium score
